# Enhanced Thrombin Generation Is Associated with Worse Left Ventricular Scarring after ST-Segment Elevation Myocardial Infarction: A Cohort Study

**DOI:** 10.3390/ph15060718

**Published:** 2022-06-06

**Authors:** Ching-Hui Sia, Sock-Hwee Tan, Siew-Pang Chan, Stephanie Marchesseau, Hui-Wen Sim, Leonardo Carvalho, Ruth Chen, Nor Hanim Mohd Amin, Alan Yean-Yip Fong, Arthur Mark Richards, Christina Yip, Mark Y. Chan

**Affiliations:** 1 Department of Cardiology, National University Heart Centre Singapore, 1E Kent Ridge Road, NUHS Tower Block Level 9, Singapore 119228, Singapore; ching_hui_sia@nuhs.edu.sg (C.-H.S.); hui_wen_sim@nuhs.edu.sg (H.-W.S.); carvalho.gt@gmail.com (L.C.); 2 Department of Medicine, Yong Loo Lin School of Medicine, National University of Singapore, 1E Kent Ridge Road, NUHS Tower Block Level 10, Singapore 119228, Singapore; mdctshw@nus.edu.sg; 3 National University Heart Centre Singapore, 1E Kent Ridge Road, NUHS Tower Block Level 9, Singapore 119228, Singapore; mdccsp@nus.edu.sg; 4 Yong Loo Lin School of Medicine, National University of Singapore, 10 Medical Drive, Singapore 117597, Singapore; 5 Savanamed, Calle Gran Vía, 30, Planta 10, 28013 Madrid, Spain; marchesseau.stephanie@gmail.com; 6 Laboratory of Genetics and Molecular Cardiology, Heart Institute (InCor-HCFMUSP), Sao Paulo 05403-904, Brazil; 7 Cardiac Department, Ferderal University of Sao Paulo State (UNIFESP), Sao Paulo 05303-000, Brazil; 8 Department of Cardiology, Woodlands Health Campus, Tower E, Level 5, Yishun Community Hospital, 2 Yishun Central 2, Singapore 768024, Singapore; chenruth@gmail.com; 9 Clinical Research Centre, Sarawak General Hospital, Jalan Hospital, Kuching 93586, Malaysia; norhanim.mohdamin@yahoo.com (N.H.M.A.); alanfong@crc.gov.my (A.Y.-Y.F.); 10 Department of Cardiology, Sarawak Heart Centre, Samarahan Expressway, Kota Samarahan 94300, Malaysia; 11 Cardiovascular Research Institute, National University of Singapore, Singapore 119228, Singapore; mark.richards@nus.edu.sg; 12 Christchurch Heart Institute, Department of Medicine, University of Otago, P.O. Box 4345, Christchurch 8140, New Zealand; 13 Department of Laboratory Medicine, Main Building, Level 3, National University Hospital, 5 Lower Kent Ridge Road, Singapore 119074, Singapore; christina_yip@nuhs.edu.sg

**Keywords:** ST-segment elevation myocardial infarction, adverse ventricular remodeling, cardiovascular magnetic resonance imaging, percutaneous coronary intervention, thrombolysis

## Abstract

Acute myocardial infarction (AMI) is associated with heightened thrombin generation. There are limited data relating to thrombin generation and left ventricular (LV) scarring and LV dilatation in post-MI LV remodeling. We studied 113 patients with ST-segment elevation myocardial infarction (STEMI) who had undergone primary percutaneous coronary intervention (PPCI) (*n* = 76) or pharmaco-invasive management (thrombolysis followed by early PCI, *n* = 37). Endogenous thrombin potential (ETP) was measured at baseline, 1 month and 6 months. Cardiovascular magnetic resonance imaging was performed at baseline and 6 months post-MI. Outcomes studied were an increase in scar change, which was defined as an increase in left ventricular infarct size of any magnitude detected by late gadolinium enhancement, adverse LV remodeling, defined as dilatation (increase) of left ventricular end-diastolic volume (LVEDV) by more than 20% and an increase in left ventricular ejection fraction (LVEF). The mean age was 55.19 ± 8.25 years and 91.2% were men. The baseline ETP was similar in the PPCI and pharmaco-invasive groups (1400.3 nM.min vs. 1334.1 nM.min, *p* = 0.473). Each 10-unit increase in baseline ETP was associated with a larger scar size (adjusted OR 1.020, 95% CI 1.002–1.037, *p* = 0.027). Baseline ETP was not associated with adverse LV remodeling or an increase in LVEF. There was no difference in scar size or adverse LV remodeling among patients undergoing PPCI vs. pharmaco-invasive management or patients receiving ticagrelor vs. clopidogrel. Enhanced thrombin generation after STEMI is associated with a subsequent increase in myocardial scarring but not LV dilatation or an increase in LVEF at 6 months post-MI.

## 1. Introduction

Ischemic heart disease is a frequent cause of death globally [1]. Myocardial infarction leads to myocardial scar formation and changes that may extend beyond the infarct border zone, causing generalized dilatation and distortion of the left ventricle (LV) [2]. This phenomenon of adverse LV remodeling is a major determinant of long-term outcomes post-ST segment elevation myocardial infarction (STEMI) as it may lead to inefficient ventricular function, heart failure and a higher risk of cardiac death [3,4]. Despite the widespread availability of evidence-based heart failure therapies such as beta-blockers and renin-angiotensin system blockers, post-STEMI admission for heart failure is still the most common cause of admission following MI [5]. There is an ongoing unmet need to identify better interventions to reduce adverse ventricular remodeling post-STEMI to prevent subsequent morbidity and mortality.

Thrombin has been implicated in post-MI scar formation and ventricular and plays an important role in post-MI pathophysiology. Thrombin participates in platelet aggregation and activates cells through its receptors leading to changes in inflammation, chemotaxis, cell growth, ventricular remodeling and myocyte contraction [6]. Intensified thrombin generation following a STEMI activates PAR-1-dependent pathways to initiate apoptosis in cardiomyocytes and promotes cardiac fibroblast proliferation and extracellular matrix deposition [7]. These pathological processes strongly determine the degree of ventricular expansion and dilatation [8]. A prothrombotic state may also participate in diastolic dysfunction due to several proposed mechanisms, such as impact on cardiac workload, endothelial function, proinflammatory cytokine expression and interaction with the renin-angiotensin-aldosterone system [9]. Previous studies have shown that both thrombolysis and percutaneous coronary intervention induce thrombin generation, although there is no direct comparison between how thrombin parameters affect left ventricular remodeling in these populations [10,11]. Higher endogenous thrombin potential (ETP) values may be present if patients have had a previous myocardial infarction [12], or in some patients, a higher body fat mass percentage [13]. Furthermore, thrombin generation in patients with acute coronary syndrome may be persistently elevated up to 24 months after the initial ACS event [14]. Theoretically, a dual-inhibition pharmacological strategy (inhibition with potent antiplatelet therapy concomitantly with anti-thrombin therapy, such as heparin) may reduce ischemia-reperfusion injury, and the consequent adverse ventricular remodeling and scarring.

The primary goal of STEMI treatment with contemporary goal-directed medical therapy is to limit infarct size and salvage ischemic myocardium, thereby preventing adverse LV remodeling which potentially leads to heart failure and cardiovascular death [15,16]. There has been interest in the development of pharmacological targets for the prevention of adverse ventricular remodeling [11,17,18,19]. The COMMANDER-HF trial showed that treatment with a factor Xa inhibitor did not improve cardiovascular outcomes of patients with chronic heart failure due to ischemic causes [20]. In contrast, the Acute Coronary Syndrome-Thrombolysis in Myocardial Infarction 51 trial (ATLAS ACS 2-TIMI 51) showed that the combination of rivaroxaban and dual antiplatelet therapy reduced cardiovascular death, myocardial infarction or stroke [21]. A subgroup analysis of patients in the ATLAS ACS 2-TIMI 51 trial who had a prior history of heart failure derived a greater benefit from thrombin suppression with rivaroxaban [21]. Notably, COMMANDER-HF excluded patients with a documented acute myocardial infarction during the index event. These two studies interpreted together suggest that a strategy of thrombin suppression may be more useful in the acute coronary syndrome context.

To investigate the feasibility of thrombin inhibition as a means of reducing myocardial infarct scar size and adverse LV remodeling, we performed a prospective sequential cardiovascular magnetic resonance (CMR) study to examine the association between thrombin generation at baseline and 1 month and the imaging endpoints of myocardial scar size, adverse ventricular remodeling, and an increase in left ventricular ejection fraction (LVEF) at 6 months after STEMI. CMR end-points have been previously shown to be associated with adverse clinical outcomes [22]. We hypothesized that heightened thrombin activity is associated with greater myocardial scarring, adverse LV remodeling and no improvement in LVEF following STEMI.

## 2. Results

### 2.1. Baseline Demographics

A total of 113 patients were recruited (Table 1). Over 90% of the patients were men and the average age was 55.19 ± 8.25 years. The baseline demographic profiles between those who received PPCI and a pharmacoinvasive strategy were similar in terms of age, hypertension, diabetes and a history of prior stroke. There were differences in the ethnic distribution mainly reflecting the demographic distribution of each site. Current smoking status was not significantly different.

In terms of laboratory findings, patients who had PPCI had a lower creatinine value compared with those who had upfront thrombolysis (84.0 umol/L vs. 94.0 umol/L, *p* = 0.017). The median troponin levels were also lower in those who had PPCI than in those who had a pharmacoinvasive strategy (Troponin I *p* < 0.001 and Troponin T *p* = 0.001). There were no significant differences in lipid profiles or HbA1c levels, nor was there any difference between the biomarkers NT-ProBNP and GDF15. Although there is a theoretical effect of thrombolysis on ETP levels [23], we found that there was no significant difference in ETP measured in the PPCI and pharmacoinvasive groups at baseline (1400.3 nM.min vs. 1334.1 nM.min, *p* = 0.473).

### 2.2. Discharge Medications

Most patients were on aspirin on discharge (99.1%) and all patients were on statins at discharge. The total proportion of patients on dual antiplatelet therapy was 99.1%. Those who received PPCI had greater ticagrelor use (PPCI 72.4% vs. pharmacoinvasive 2.7%) while those who had a pharmacoinvasive strategy received more clopidogrel (PPCI 27.6% vs. pharmacoinvasive 97.3%) (combined *p* < 0.001). No patient received prasugrel. Patients with PPCI also had a higher rate of beta-blocker use at discharge (PPCI 84.2% vs. pharmacoinvasive 64.9%, *p* = 0.020), although there was no statistically significant difference in the use of ACE-I/ARB (68.4% PPCI vs. 54.1% pharmacoinvasive, *p* = 0.136).

### 2.3. Thrombin Parameters

**Figure 1:**
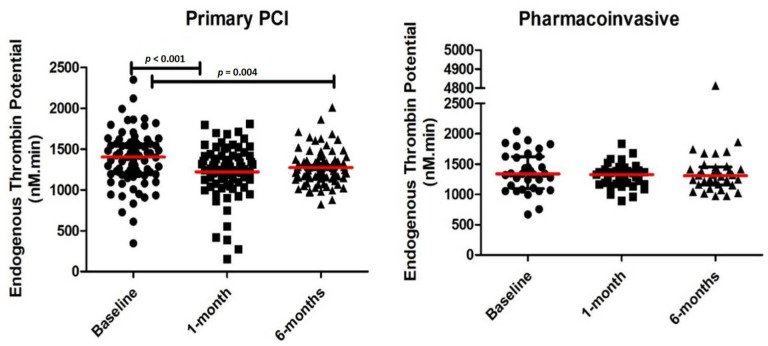
Endogenous thrombin potential (ETP) levels at baseline, 1-month and 6-months in patients receiving primary percutaneous coronary intervention (PCI) and pharmacoinvasive therapy.

We compared the temporal trend in thrombin parameters from baseline to 1 and 6 months (Figure 1 and Table 2). There was a significant decrease in ETP from baseline at 1 and 6 months in the PPCI group. However, there was no significant change in ETP from baseline at 1 and 6 months in the pharmacoinvasive group.

### 2.4. CMR Results

A total of 31 (40.8%) patients in the PPCI group and 12 (32.4%) patients in the pharmacoinvasive group met the endpoint of scar size increase. A total of 15 patients met the predefined endpoint for adverse ventricular remodeling (9.7%), with 7 (9.2%) in the PPCI group and 4 (10.8%) in the pharmacoinvasive group (*p* = 0.734) (Table 3). Of the various cardiac chamber parameters examined, significant differences were detected only in the PPCI group for LVESV which fell from 48.74 mL at baseline to 39.41 mL at 6 months (*p* < 0.001) and for LVEF, which rose from 57.07% to 63.42% (*p* < 0.001).

### 2.5. Relation between ETP and Imaging Endpoints

As illustrated in Table 4, each 10-unit increase in baseline ETP was associated with a larger scar size (adjusted OR 1.019, 95% CI 1.002–1.036, *p* = 0.027). Current smoking status, but not ex-smoking status, showed a protective association against increased scar size (adjusted OR 0.311, 95% CI 0.105–0.919, *p* = 0.035). Results on adverse remodeling (>20% increase in LVEDV) are depicted in Table 5 and results on increase in LVEF are depicted in Table 6. ETP measured at baseline did not predict adverse LV remodeling (adjusted OR 0.986, 95% CI 0.962–1.010, *p* = 0.248) or an increase in LVEF (adjusted OR 0.993, 95% CI 0.977–1.009, *p* = 0.378). Whether thrombolysis was performed affected neither the scar size (adjusted OR 1.064, 95% CI 0.227–4.992, *p* = 0.938), adverse ventricular remodeling (adjusted OR 0.581, 95% CI 0.033–10.320, *p* = 0.712) nor an increase in LVEF (adjusted OR 1.194, 95% CI 0.263–5.426, *p* = 0.819). Use of ticagrelor as compared to clopidogrel was not associated a change in scar size (adjusted OR 1.481, 95% CI 0.383–5.731, *p* = 0.569), adverse ventricular remodeling (adjusted OR 2.265, 95% CI 0.266–19.307, *p* = 0.455) nor an increase in LVEF (adjusted OR 3.378, 95% CI 0.806–14.150, *p* = 0.096). 

## 3. Materials & Methods

### 3.1. Study Design and Population

**Figure 2:**
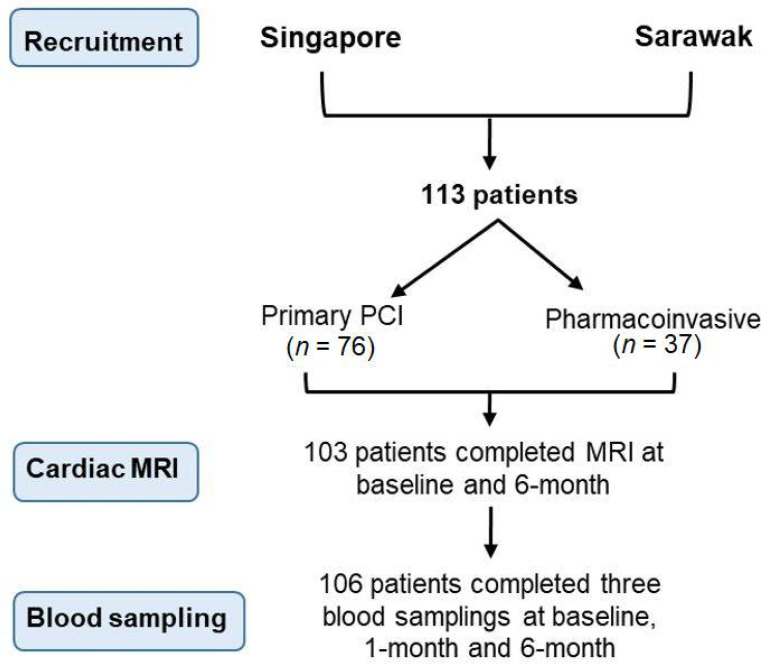
Flow diagram describing the patients recruited in the study.

We conducted a prospective, dual-center, bioimaging study to investigate the longitudinal relationship between persistent thrombin activity and adverse ventricular remodeling. The National University Heart Centre Singapore, Singapore (NUHCS) and Sarawak General Hospital Heart Centre, Malaysia (SGHHC) participated in this study from July 2015 to September 2017 (Figure 2). STEMI patients at NUHCS were primarily treated with PPCI while patients from SGHHC typically had more thrombolytic use as the initial reperfusion strategy, followed by early percutaneous coronary intervention (pharmaco-invasive strategy). We included both female and male patients aged ≥21 and ≤85 years old, with a diagnosis of STEMI within 14 days from the index event, who were able to provide informed consent. Study participants could be enrolled immediately after the informed consent was given.

STEMI was defined in accordance with the third universal definition of myocardial infarction: elevation of the ST-segment of more than 0.1 millivolts (mv) in 2 or more contiguous electrocardiogram leads, or a new left bundle branch block, or ST-segment depression 0.1 mV or greater in 2 of the precordial leads V1–V4 with evidence suggestive of a true posterior MI, with elevated biomarkers of myocardial necrosis (troponin, creatine kinase-muscle brain (CK-MB) isoenzyme) [24].

Exclusion criteria were decided based on ability to comply with study protocols, ability to perform CMR and factors that would affect thrombin levels. The exclusion criteria were inability to provide consent, inability to comply with the follow-up protocol, anemia (defined as a hemoglobin level <8 g/dL in men and <7 g/dL in women), a history of significant valvular heart disease (defined as patients with a valvular pathology graded moderate and above in severity) [25], a history of malignancy diagnosed within the last 12 months [26], use of immunosuppressive therapy [27], contraindications to CMR (e.g., claustrophobia, non-magnetic resonance imaging compatible metallic implants or cardiac implantable electronic devices), indications for chronic anticoagulation (e.g., atrial fibrillation, prosthetic heart valves, deep vein thrombosis), on anticoagulants within 24 h of the blood draw and renal impairment with an estimated glomerular filtration rate of <60 mL/min/m^2^. The eligible participants were enrolled consecutively from patients undergoing coronary angiography once they had satisfied the above criteria and had consented to the study.

### 3.2. Study Procedures and Follow-Up

All patient data were documented at the initial study visit and thereafter at 1- and 6-monthly intervals after study enrollment. During the initial visit, a clinical assessment was done, including routine treatment data such as laboratory investigations (Troponin I, Troponin T, GDF-15 and NT-ProBNP) as well as the use of concomitant medication.

### 3.3. Laboratory Testing

Blood samples were obtained from patients via venipuncture into a 3.2% sodium citrate vacutainer tube (Becton Dickinson Vacutainer, Franklin Lakes, NJ, USA) within 48–72 h of admission and subsequently at 1- and 6-months post-discharge. We measured calibrated automated thrombograms using thrombin-specific and Xa-specific chromogenic substrates, to monitor the degree of thrombin generation after STEMI, at baseline, 1 month, and at 6 months. Citrated whole blood was centrifuged at 2500× *g* for 10 min to obtain platelet-poor plasma (PPP). The plasma was stored for 8 h at 20 ± 5 °C or stored within 4 h of collection at −80 °C. Frozen stored samples were thawed at 37 °C for 10 min before testing.

### 3.4. Thrombin Generation Measurements

The thrombin generation assay was performed using the Calibrated Automated Thrombography (CAT, Diagnostica Stago, Asnières-sur-Seine, France) according to the manufacturer’s standard procedure. Briefly, 20 µL of starting reagent (containing tissue factor at final concentration of 5 pM) or thrombin calibrator was added to each test well of a 96-well round-bottom microtiter plate (Nunc, Denmark). 80 µL of PPP was added to the test wells and the microtiter plate was then warmed up at 37 °C for 5 min in the CAT instrument. The reaction is initiated when 20 µL of fluorescence substrate/CaCl_2_ mixture was dispensed into each well. During the 60 min of reaction time, the fluorescence was measured (excitation filter at 390 nm and emission filter at 460 nm) at 20-s intervals. Each assay was performed as triplicate and ETP was calculated by the thrombinoscope software (Diagnostica Stago, Asnières-sur-Seine, France). Numerical values and traces of thrombography were retained for result analysis. ETP represents the total amount of thrombin a plasma sample could generate under pro- and anti-coagulant factors operating simultaneously in the plasma [28].

### 3.5. Cardiac Magnetic Resonance Imaging

MR imaging was performed at baseline and at 6 months at both centers. Images were acquired in two sites using different protocols of acquisition. In Singapore, the acquisition was performed on a 3T Biograph mMR (Siemens, Munich, Germany) and a 3T Prisma MR (Siemens) depending on scanner availability. The full CMR imaging protocol included a breath-hold CINE steady-state free precession (SSFP) short-axis covering both the right ventricle (RV) and the left ventricle (LV) with a slice thickness of 8.5 mm and a 20% gap with an image resolution of 1.33 × 1.33 × 10 mm. Late gadolinium enhancement (LGE) short-axis images were acquired 10 min after a 0.2 mmol/kg GADOVIST injection. Sequential parameters of the LGE acquisition were the following: TR = 4.1 ms, Average R-R interval = 990 ms, TE = 1.56 ms, Slice thickness = 8.5 mm, Gap = 20%, Flip angle = 20, Inversion time = subject-specific calculated using TI scout, Image resolution = 1.33 mm × 1.33 mm × 10 mm. In Sarawak, the acquisition was performed on a 1.5T Achieva MR (Philips, Amsterdam, The Netherlands). The CINE sequences were acquired using a breath-hold CINE balanced SSFP short-axis covering the whole heart with a slice thickness of 8 mm and no gap, thus leading to an image resolution of 1.33 mm × 1.33 mm × 8 mm. LGE short-axis images matching the cine stack were acquired 10 min after injection of 0.2 mmol/kg of GADOVIST using a PSIR SENSE sequence with the following parameters: TR = 6.1 ms, Average R-R interval = 1000 ms, TE = 2.99 ms, Flip angle = 25, Inversion time = subject-specific calculated using TI scout with an image resolution of 1.34 mm × 1.34 mm × 8 mm. All sequences were analyzed automatically using a deep learning pipeline that has been published previously [29], regardless of the original hospital or scanner. After some pre-processing (resizing all images to the same resolution), each sequence was segmented using a specific U-Net [30] model which was previously trained on an independent dataset and extensively validated, showing high robustness and accuracy higher than intra-observer reproducibility. After the automatic analysis, a subset of images (30%) was randomly selected for manual corrections. Manual corrections were performed using in-house software designed for this purpose, and manual parameters were extracted for comparison with the automatic ones. Confidence in the measured parameters could then be assessed statistically in terms of Pearson’s correlation and mean absolute error. Investigators interpreting the CMR, and coagulation studies were blinded to the study subject data.

### 3.6. Statistical Analysis

The data were reported as mean ± standard deviation or median (interquartile range) for quantitative data, and frequency (%) for qualitative data. The Wilcoxon signed-rank test and the chi-square test were applied for ascertaining if there was a group difference (PPCI vs. pharmacoinvasive). The statistical plan considered the two groups separately as we expected an effect of thrombolysis on thrombin levels [31]. The confirmatory analyses concerning a binary end-point of infarct size increase from baseline (increase or no increase) measured by LGE, adverse ventricular remodeling (i.e., >20% increase of left ventricular end-diastolic volume [LVEDV] from baseline) [32] or an increase of left ventricular ejection fraction (LVEF) from baseline (increase or on increase) were performed with a mixed model within the structural equation modeling framework, which took into account the sequential nature of endogenous thrombin potential at baseline, 1 month and 6 months. Given that the outcomes were binary, the chosen underlying probability distribution and link function were Binomial and logit respectively. The considered predictors included age, smoking status (non/current/former smoker) and comorbid conditions (hypertension, diabetes and previous stroke). These covariates were included in view of their clinical importance. Adjustment for sex was not performed in view of the overwhelming majority of male subjects (91.2%). Anticipating that the thrombin parameters could be correlated instantaneously and longitudinally, care was taken to identify the final model. While a backward elimination (removal *p* > 0.05) was implemented in model-building, other criteria such as interpretability were also considered. The data were complete, except for LVEF (the proportion of missing cases was <10%). No imputation was performed for missing data. The data were analyzed with Stata MP V16 (StataCorp, Universal City, TX, USA) and all statistical tests were conducted at a 5% level of significance.

### 3.7. Ethics Approval

The study was conducted in accordance with the principles of the Declaration of Helsinki and ethics approval was obtained from the respective institutional review boards of the institutions [National University Heart Centre Singapore—NHG DSRB 2013/00248 under the Post-Acute MI Left ventricular remodeling biomarker analysis [PAMILA] study; the TIRAMI sub-study as 2013/00248-AMD0002 and the Sarawak General Hospital Heart Centre, Malaysia Medical Research and Ethics Committee NMRR_14-1165-22331(IIR)].

## 4. Discussion

In this cohort of patients with STEMI, a higher ETP at baseline was associated with an increase in LV scar size but not adverse ventricular remodeling (dilatation) or an increase in LVEF over 6 months. These associations were consistent regardless of whether patients received upfront PPCI or pharmacoinvasive management. We further observed an association between current cigarette smoking and a decrease in LV scar size, a finding that is consistent with other studies suggesting a protective effect of cigarette smoking on infarct size [33]. Interestingly, more potent platelet P2Y12 inhibition was not associated with a reduction in LV scar size. The results from this study suggest that a dual-pathway inhibition strategy with P2Y12 inhibition and thrombin inhibition could be explored as a means to reduce myocardial scar formation in the early post-STEMI period.

Our study provides mechanistic insight into how ETP correlates with post-MI myocardial scarring. There is potential in the strategy of suppressing thrombin generation with anticoagulation, and this may improve clinical outcomes by leading to a reduction in myocardial scar size. In the heart failure population as in the trial COMMANDER-HF, the scar would have already been formed and thrombin suppression may be too little too late in the disease spectrum to prevent adverse cardiovascular outcomes. The use of thrombin-inhibitors may be a reasonable strategy given that there was no increase in bleeding with the use of low-dose rivaroxaban as compared to aspirin in the GEMINI-ACS-1 trial [34]. Preliminary data from a murine model studying the use of anti-thrombin nanoparticles limits ischemia-reperfusion injury and as such anti-thrombin agents may have a role in reducing myocardial scarring [35].

Although the PPCI subgroup had a significant decrease in the levels of ETP compared to the pharmacoinvasive group over a 6-month period, which suggests that PPCI is preferred in reducing thrombin generation and subsequent scarring of the myocardium as compared to a pharmacoinvasive strategy, we actually found that there was a similar infarct size at 6 months regardless of whether patients underwent a PPCI or pharmacoinvasive strategy. We did not find any effect of thrombolysis on the effect of scar size on adjusted analysis, but it might be worth exploring the differences in antiplatelet strategies in post-PCI and post-thrombolysis patients and if thrombin suppression leads to different outcomes in these patients. Sonin et al. studied the use of a PAR-1 inhibitor by SCH 79797 in a rat model of ischemia-reperfusion injury. They found that PAR-1 inhibition attenuated adverse LV remodeling, together with a non-significant reduction in scar size. Direct thrombin inhibitors have been demonstrated to prevent left atrial remodeling in a murine model of heart failure [36]. Additionally, in a murine model, direct thrombin inhibition has also been shown to reduce pressure overload-induced cardiac fibrosis [37]. One caveat is that the strategy of dual pathway inhibition with antiplatelets and anti-thrombin agents may need to be examined carefully for bleeding risk in thrombolysis patients, as the aforementioned GEMINI-ACS-1 trial consisted mainly of PPCI patients and may not be directly extrapolatable to the thrombolysis population [34]. More studies are needed on the population receiving thrombolysis, a treatment still used for many patients in developing countries and rural areas [38,39]. As such, any consideration for a dual-pathway inhibition strategy needs to be tailored to patients with PPCI or thrombolysis.

The choice of antiplatelet medications in our study population deserves mention. We analyzed the effect of the use of clopidogrel vs. ticagrelor in our regression model. The PLATO trial showed that at 12 months, the death rate from myocardial infarction, vascular causes or stroke was reduced in patients randomized to ticagrelor as compared to clopidogrel [40]. We expected a similar trend in our patients, hypothesizing that the use of the potent P2Y12 inhibitor ticagrelor would lead to reduced scarring over clopidogrel as well as a reduced incidence of LV adverse remodeling. Instead, we found no association between choice of agent and scar size or adverse LV remodeling. Rather, the ETP level is associated with scar size. This suggests that thrombin-inhibition may be a focus for reducing myocardial scarring in STEMI patients. Thrombin-inhibition may be more beneficial than antiplatelet therapy in exerting beneficial effects on the myocardium as compared to antiplatelet therapy which acts primarily to reduce vascular events.

Current smoking status was significantly associated with reduced odds of myocardial scarring. The ‘smoker paradox’ has previously been described, where smokers were found to have better cardiovascular outcomes. A study in ST-segment elevation myocardial infarction patients who underwent primary percutaneous coronary intervention showed a lower adjusted risk of in-hospital mortality in smokers [41]. However, reports from imaging studies conflict with regard to whether smoking is associated with myocardial scarring [42,43]. Our study demonstrated that current smokers, but not ex-smokers, had a smaller myocardial scar size on CMR at 6-months. As such, there appear to be conflicting results in terms of smoking and its associated post-MI epidemiological and imaging outcomes. One possible explanation is that our study might not have taken into account unidentified confounders that could have falsely led to the interpretation of a ‘smoker paradox’, as has been shown in previous epidemiological studies where such an apparent paradox disappeared after adjustment for confounding [43]. We adjusted for known confounders but cannot account for possible as yet unidentified confounders. In addition, smoking may promote coronary collateral formation and facilitate ischemic pre-conditioning of the myocardium, leading to a protective effect [44,45]. However, given the overwhelmingly negative effects of tobacco smoking on overall health [46], prudence is advised when interpreting this finding.

Our study has several limitations. This was an observational study and as such we could only determine association but not causality in the relation between heightened baseline thrombin generation and subsequent myocardial scar size. Enrolling both PPCI and pharmacoinvasive STEMI patients meant that the sample size for each subgroup was reduced; however, we decided that it was necessary to enroll both subgroups of STEMI patients to enable better generalizability of our study results as a large proportion of patients globally still receive fibrinolytic treatment as the initial reperfusion modality [47]. A majority of patients in this study were male, hence future studies should also focus on female patients to ensure the applicability of these results to the female population. Larger studies will need to be performed to confirm the findings from this study given the small sample size. We also did not have D-dimer results for this patient cohort, and that needs to be studied in the future [48]. Finally, this was not an interventional study so we were unable to determine if suppression of thrombin generation using anticoagulant therapy could reduce subsequent myocardial scar size.

## 5. Conclusions

A higher ETP at baseline was associated with an increased myocardial scar size but not LV dilatation or a change in LVEF over 6 months, regardless of a PPCI or pharmaco-invasive strategy. These data suggest that a clinical trial of dual pathway inhibition in the STEMI population may be warranted to reduce myocardial scar size among patients with enhanced thrombin generation parameters.

## Figures and Tables

**Table 1:** Baseline characteristics of study population.

	Total (*n* = 113)	Primary PCI (*n* = 76)	Pharmacoinvasive (*n* = 37)	*p*-Value
Mean age (years)	55.19 ± 8.25	55.97 ± 7.69	53.57 ± 9.19	0.146
Male (%)	103 (91.2)	72 (94.7)	31 (83.8)	0.054
Ethnicity (%) Chinese Malay Indian Others	49 (43.4) 35 (31.0) 11 (9.7) 18 (15.9)	42 (55.3) 16 (21.1) 11 (14.5) 7 (9.2)	7 (18.9) 19 (51.4) 0 (0.0) 11 (29.7)	<0.001
Smoking status Current Ex-smoker Never smoker	66 (58.4) 12 (10.6) 35 (31.0)	42 (55.3) 7 (9.2) 27 (35.5)	24 (64.9) 5 (13.5) 8 (21.6)	0.304
History of diabetes	26 (23.0)	17 (22.4)	9 (24.3)	0.817
History of hypertension	54 (47.8)	41 (53.9)	13 (35.1)	0.060
History of hyperlipidaemia	49 (43.4)	35 (46.1)	14 (37.8)	0.408
Family history of premature CAD	15 (13.3)	8 (10.5)	7 (18.9)	0.217
Prior MI	3 (2.7)	2 (2.6)	1 (2.7)	0.982
Prior PCI	2 (1.8)	1 (1.3)	1 (2.7)	0.600
Prior CABG	0 (0.0)	0 (0.0)	0 (0.0)	N/A
Prior stroke	4 (3.5)	4 (5.3)	0 (0.0)	0.155
**Laboratory Parameters**				
Total cholesterol (mmol/dL)	5.33 (4.61, 5.87)	5.26 (4.65, 5.68)	5.40 (4.50, 5.90)	0.503
Triglyceride (mmol/dL)	1.59 (1.16, 2.29)	1.51 (1.16, 2.22)	1.77 (1.22, 2.50)	0.230
Low density lipoprotein cholesterol (mmol/dL)	3.43 (2.70, 4.00)	3.50 (2.63, 4.00)	3.40 (2.95, 4.05)	0.752
High density lipoprotein (mmol/dL)	1.06 (0.96, 1.20)	1.08 (0.96, 1.22)	1.03 (0.93, 1.17)	0.222
HbA1c (%)	6.1 (5.7, 7.0)	5.95 (5.65, 6.95)	6.75 (6.10, 10.95)	0.127
Creatinine (umol/L)	87.0 (77.0, 102.0)	84.0 (75.0, 98.5)	94.0 (80.0, 108.0)	0.017
Troponin I (ng/mL)	14,492.1 (1787.7, 38,633.0)	11,311.6 (913.1, 26,518.0)	30,641 (14,233.2, 46,625.2)	<0.001
Troponin T (ng/mL)	2086.0 (600.1, 3898.0)	1581.5 (395.1, 3349.5)	3054.0 (1816.0, 5478.0)	0.001
NT-ProBNP	831.1 (493.6, 1463.0)	822.2 (530.4, 1469.0)	899.5 (327.3, 1463.0)	0.920
GDF15	1278.0 (926.0, 1778.0)	1298.0 (902.0, 1787.0)	1252.5 (929.4, 1771.0)	0.882
White cell count (10^9^/L)	11.3 (9.6, 13.9)	11.2 (9.3, 12.9)	12.1 (9.9, 14.0)	0.127
Platelet count (10^9^/L)	249.0 (214.0, 293.0)	250.5 (215.0, 296.5)	244.0 (200.0, 280.0)	0.305
Haemoglobin (10^9^/L)	15.1 (13.9, 15.7)	15.1 (14.0, 15.7)	14.7 (13.9, 15.7)	0.825
Endogenous Thrombin Potential (*n* = 104) (nM.min)	1355.7 (1170.1, 1570.3)	1400.3 (1190.6, 1565.2)	1334.1 (1079.5, 1605.4)	0.473
**Discharge Medications**				
Aspirin	112 (99.1)	75 (98.7)	37 (100.0)	0.483
P2Y12 Inhibitor Clopidogrel Ticagrelor	57 (50.4) 56 (49.6)	21 (27.6) 55 (72.4)	36 (97.3) 1 (2.7)	<0.001
Statin	113 (100.0)	76 (100.0)	37 (100.0)	N/A
Beta-blocker	88 (77.9)	64 (84.2)	24 (64.9)	0.020
ACE-I/ARB	72 (63.7)	52 (68.4)	20 (54.1)	0.136

**Table 2:** Baseline and follow-up thrombin generation parameters.

Variable	Primary PCI			Pharmacoinvasive		
	Baseline	1 Month	6 Months	Baseline	1 Month	6 Months
Endogenous Thrombin Potential (nM.min)	1400.29 (1190.57, 1565.19)	1261.15 (1110.84, 1419.88) *	1248.01 (1139.72, 1368.45) **	1334.14 (1079.45, 1605.42)	1326.52 (1173.72, 1413.01)	1307.73 (1161.83, 1448.04)

* Significant difference between 1 month and baseline (*p* < 0.001). ** Significant difference between 6 months and baseline (*p* = 0.004).

**Table 3:** Magnetic resonance imaging parameters at baseline and at 6-months for the primary PCI and pharmacoinvasive groups.

Variable	Primary PCI				Pharmacoinvasive				
	Baseline	6 Months	Change	*p* Value Comparing Baseline and 6 Months	Baseline	6 Months	Change	*p* Value Comparing Baseline and 6 Months	*p* Value Comparing Change in Value of PPCI and Pharmacoinvasive Groups
Infarct Size (LGE) (%)	21.68 (14.41, 26.59)	19.96 (14.48, 24.62)	−0.76 (−7.21, 3.31)	0.251	16.60 (12.88, 21.68)	15.34 (9.45, 22.92)	−2.88 (−5.46, 2.17)	0.106	0.680
Adverse ventricular remodeling *	-	7 (10.9)		-	-	4 (13.8)		-	
LVESV (mL)	48.74 (38.07, 69.39)	39.41 (28.98, 56.77)	−7.07 (−16.05, 0.58)	<0.001	55.48 (40.57, 71.48)	48.18 (34.96, 72.27)	−3.87 (−9.83, 6.89)	0.388	0.081
LVEDV (mL)	117.18 (103.75, 137.94)	114.22 (97.27, 137.23)	0.56 (−13.58, 13.02)	0.863	129.20 (111.99, 148.14)	123.06 (111.17, 149.21)	−1.69 (−9.38, 13.14)	0.992	0.885
LVEF (%)	57.07 (47.48, 65.17)	63.42 (56.84, 72.36)	5.66 (1.68, 11.29)	<0.001	55.20 (49.33, 65.59)	59.33 (48.82, 67.34)	2.37 (−3.06, 5.56)	0.337	0.005

Continuous variables are presented as median with interquartile ranges, while categorical variables are presented as numbers and proportions. Abbreviations: LGE, late gadolinium enhancement; LVEDV, left ventricular end-diastolic volume; LVEF, left ventricular ejection fraction; LVESV, left ventricular end-systolic volume. * Adverse ventricular remodeling is defined as a >20% change in LVEDV.

**Table 4:** Predictors of scar increase measured by late gadolinium enhancement on cardiac magnetic resonance imaging.

Variable	Adjusted Odds Ratio	95% CI	*p* Value
ETP Base (10 units)	1.019	1.002–1.036	0.027
Age	0.953	0.892–1.018	0.154
Hypertension	0.504	0.177–1.436	0.200
Diabetes mellitus	1.398	0.435–4.496	0.574
Smoking Status Current Ex-smoker	0.311 0.203	0.105–0.919 0.028–1.482	0.035 0.116
Previous stroke	2.185	0.170–28.062	0.548
Primary PCI Pharmacoinvasive	Reference 1.064	0.227–4.992	0.938
Clopidogrel Ticagrelor	Reference 1.481	0.383–5.731	0.569

Abbreviations: CI, confidence interval; ETP, endogenous thrombin potential; PCI, percutaneous coronary intervention.

**Table 5:** Predictors of adverse ventricular remodeling (≥20% increase of left ventricular end-diastolic volume) outcome.

Variable	Adjusted Odds Ratio	95% CI	*p* Value
ETP base (10 units)	0.986	0.962–1.010	0.248
Age	1.012	0.917–1.117	0.806
Hypertension	2.432	0.483–12.247	0.281
Diabetes mellitus	1.885	0.419–8.493	0.409
Smoking Status Current Ex-smoker	0.750 2.984	0.173–3.249 0.260–34.170	0.700 0.380
Previous stroke	2.626	0.212–32.553	0.452
Primary PCI Pharmacoinvasive	Reference 0.581	0.033–10.320	0.712
Clopidogrel Ticagrelor	Reference 2.265	0.266–19.307	0.455

Abbreviations: CI, confidence interval; ETP, endogenous thrombin potential; PCI, percutaneous coronary intervention.

**Table 6:** Predictors of increase in left ventricular ejection fraction outcome.

Variable	Adjusted Odds Ratio	95% CI	*p* Value
ETP base (10 units)	0.993	0.977–1.009	0.378
Age	1.005	0.939–1.075	0.890
Hypertension	1.691	0.533–5.364	0.372
Diabetes mellitus	0.445	0.131–1.512	0.195
Smoking Status Current Ex-smoker	0.766 2.392	0.226–2.592 0.181–31.601	0.668 0.508
Primary PCI Pharmacoinvasive	Reference 1.194	0.263–5.426	0.819
Clopidogrel Ticagrelor	Reference 3.378	0.806–14.150	0.096

Abbreviations: CI, confidence interval; ETP, endogenous thrombin potential; PCI, percutaneous coronary intervention.

## Data Availability

The datasets used and/or analyzed during the current study are available from the corresponding author on reasonable request.
